# Communication barriers in maternity care of allophone migrants: Experiences of women, healthcare professionals, and intercultural interpreters

**DOI:** 10.1111/jan.14093

**Published:** 2019-06-21

**Authors:** Paola Origlia Ikhilor, Gabriele Hasenberg, Elisabeth Kurth, Fana Asefaw, Jessica Pehlke‐Milde, Eva Cignacco

**Affiliations:** ^1^ Department of Health Professions Bern University of Applied Sciences Bern Switzerland; ^2^ Institute of Midwifery Zurich University of Applied Sciences Winterthur Switzerland; ^3^ Swiss Tropical and Public Health Institute, University of Basel Basel Switzerland; ^4^ Familystart of both Basel Basel Switzerland; ^5^ Outpatient Clinic for Child and Adolescent Psychiatry and Psychotherapy Clienia Littenheid AG Winterthur Switzerland

**Keywords:** access to health care, communication barriers, health services needs and demand, intercultural interpreter, maternal health services, midwifery, migrants, nursing, professional–patient relations

## Abstract

**Aim:**

To describe communication barriers faced by allophone migrant women in maternity care provision from the perspectives of migrant women, healthcare professionals, and intercultural interpreters.

**Background:**

Perinatal health inequality of migrant women hinges on barriers to services, with a major barrier being language. Their care is often also perceived as demanding due to conflicting values or complex situations. Potentially divergent perceptions of users and providers may hinder efficient communication.

**Design:**

Qualitative explorative study.

**Methods:**

A convenience sample of 36 participants was recruited in the German speaking region of Switzerland. The sample consisted of four Albanian and six Tigrinya speaking women, 22 healthcare professionals and four intercultural interpreters (March–June 2016) who participated in three focus group discussions and seven semi‐structured interviews. Audio recordings of the discussions and interviews were transcribed and thematically analysed.

**Results:**

The analysis revealed three main themes: the challenge of understanding each other's world, communication breakdowns and imposed health services. Without interpretation communication was reduced to a bare minimum and thus insufficient to adequately inform women about treatment and address their expectations and needs.

**Conclusion:**

A primary step in dismantling barriers is guaranteed intercultural interpreting services. Additionally, healthcare professionals need to continuously develop and reflect on their transcultural communication. Institutions must enable professionals to respond flexibly to allophone women's needs and to offer care options that are safe and in accordance to their cultural values.

**Impact:**

Our results give the foundation of tenable care of allophonic women and emphasize the importance of linguistic understanding in care quality.

## Abstract (zh)

**目的:**

从移民妇女、护理专业人员和跨文化口译员的角度,描述移民妇女在接受孕产妇护理方面面临的沟通障碍。

**背景:**

移民妇女的围产期护理不平等来自于服务障碍,其中一个主要障碍是语言。由于价值观的冲突或复杂的情况,人们认为她们的护理要求很高。对用户和提供者潜在的不同的看法可能会妨碍有效的沟通。

**设计:**

定性的探索性研究。

**方法:**

选取瑞士德语区的36名受试者为便利样本。其中包括4名阿尔巴尼亚人和6名讲提格里尼亚语的妇女、22名医疗护理专业人员和4名跨文化口译员(2016年3月至6月),她们参加了3次专题小组讨论和7次半结构式访谈。对讨论和访谈的录音进行了转录和主题分析。

**结果:**

分析揭示了三个主要主题:相互了解世界的挑战、沟通障碍和强制医疗服务。如果没有口译,沟通就被减少到最低限度,因此不足以向妇女充分介绍治疗情况,难以满足她们的期望和需要。

**结论:**

消除障碍的首要任务是确保跨文化口译服务。此外,医疗护理专业人员需要不断发展和反思他们的跨文化交流。机构必须使专业人员能够灵活地满足妇女的各种需要,并提供安全和符合其文化价值观的护理选择。

**影响:**

我们的结果为不同语言的女性提供优质护理打下了基础,强调语言理解对护理质量的重要性。

## 1 INTRODUCTION

There is incontrovertible evidence that migrant women suffer from more maternal and general health problems than native‐born women (Bundesamt für Gesundheit, 2012; Keygnaert et al., 2014; Merten & Gari, 2013). The poorer health of migrants has numerous causes. Notably, refugees may have pre‐existing health problems resulting from inadequate healthcare services in their home countries or suffer from privation or traumas experienced during their flight. Furthermore, poorer perinatal outcome is also associated with a low socioeconomic status (de Graaf, Steegers, & Bonsel, 2013; Vos, Posthumus, Bonsel, Steegers, & Denktas, 2014). Factors such as poor living conditions, unemployment, and poverty adversely influence migrant maternal health. The health inequality of pregnant migrants and their newborns does however seem to be primarily related to difficultly accessing perinatal care services in the host country (Bundesamt für Gesundheit, 2013; Cignacco et al., 2018; Keygnaert et al., 2016; Merten & Gari, 2013). It has also been shown that there is a direct link between language barriers and adverse birth outcomes (Sentell, Chang, Ahn, & Miyamura, 2016).

### 1.1 Background

Access to health care is considered the ability to identify healthcare needs, seek and reach healthcare services, to obtain and use healthcare, and to have those health needs addressed. Healthcare access results from complex interaction of healthcare systems, organizations and providers with the characteristics of individuals and their social and physical environments (Levesque, Harris, & Russell, 2013).

In Switzerland, maternity care is generally given during pregnancy by obstetricians, then during birth and early postpartum by interdisciplinary teams in hospitals, followed by postpartum at home by midwives and finally, for children up to 1 year by community‐based maternity counsellors and paediatricians. A range of other maternity care services and providers complement this standard care, such as antenatal classes, childbirth, and postnatal care in a birth centre or at home, breastfeeding counselling (Navarra & Liewald, 2017). Most of these costs are covered by health insurance. Though the standard of care can be considered high, maternity care services are highly fragmented which may affect migrant women's ability to identify and reach them, thus hindering continuous maternity care provision (König & Pehlke‐Milde, 2010).

About 30% of the children born in Switzerland are foreign and 40% have a foreign born mother (Federal Statistical Office [Bundesmat für Statistik BFS], 2018a). Of all documented foreign women who have lived in Switzerland for more than a year, 11.2% do not speak an official language, this number rises to a 25% amongst first‐generation non‐European nationals (BFS, 2018b). In addition, migrants such as asylum seekers and undocumented foreigners rarely speak a Swiss language. Therefore, healthcare professionals serve a considerable number of allophone migrant women and thus are affected by linguistic barriers which impede effective and equitable health care. We define allophone migrants as migrants who do not speak any of the official languages of their host country.

Research on migrant women's experiences of maternity illustrates that allophone women face many barriers accessing these services. These barriers include lack of awareness of their existence, insufficient support accessing maternity services and discordant expectations between the women and service providers (Higginbottom, Bell, Arsenault, & Pillay, 2012). Moreover, caring for migrant women is perceived as demanding because of their often complex socioeconomic or asylum‐related problems. Care providers often observe this group's lack of health literacy and knowledge of the maternity care system. Language barriers and conflicting values further complicate their treatment and care (Boerleider, Francke, Mannien, Wiegers, & Deville, 2013; Kurth, 2013; Pelaez, Hendricks, Merry, & Gagnon, 2017).

Intercultural interpreting effectively ensures mutual understanding and has been shown to positively affect health (Gehrig, Caldéron, Guggisberg, & Gardiol, 2012). In addition to standard translation, intercultural interpretation requires a sound knowledge of the migrant group's socio‐cultural frameworks, circumstances and the local structures. Intercultural interpreters are able to move in both the “foreign” and “local” frames of reference and relate them to each other (Interpret, 2017). However, in Switzerland intercultural interpreters’ frequency of deployment varies in hospitals since the cost of interpretation services is not regulated. In outpatient treatment it is even more difficult to afford interpreting services.

Healthcare delivery fundamentally depends on effective and efficient communication (Vermeir et al., 2015). The evidence reviewed shows that both, users and providers experience barriers that may hinder it. Juxtaposing their experiences and including the view from interpreters as key informants, could reveal divergent perceptions and allow formulating appropriate culturally acceptable measures.

## 2 THE STUDY

### 2.1 Aim/s

This study aimed to describe the communication barriers faced by allophone migrant women when accessing maternity care service. We explored the perspectives of maternity service users, healthcare professionals and intercultural interpreters in the German speaking part of Switzerland. The findings served as a basis for recommendations to improve quality and access to maternity care services.

### 2.2 Design

This exploratory, qualitative study was designed to reveal processes, interpretive frames, and patterns of communication barriers from multiple perspectives using focus group discussions (FGD; Krueger & Casey, 2009) and problem‐centred interviews (Witzel, 2000). It was part of a broader investigation into allophone migrant women's access to maternity care (Origlia Ikhilor et al., 2017); detailed information on that study's methodology is described there. In the study for this article, we used the consolidated criteria framework for reporting qualitative studies (COREQ; Tong, Craig, & Sainsbury, 2007). The COREQ checklist is available as supplementary online material.

### 2.3 Participants

Convenience sampling was used to recruit a total of 36 individuals: users, healthcare professionals, and intercultural interpreters (Tables 1 and 2). The group of users was composed of six Eritrean, one Albanian and three Kosovan mothers. They had between one and four children each, with the youngest child's average age being 18 months. They had all been treated in the German‐speaking part of Switzerland during pregnancy, birth, and postpartum. Half of the women understood simple sentences in German, the other half only few words or no German at all. Women with serious complications during their most recent pregnancy, childbirth or postpartum were not eligible to take part in our study.

**Table 1:** Age of participants, age of youngest infant, and residence duration of users (*N* = 36)

Characteristics	Range	Mean	Median	*SD*
Healthcare professionals (*n* = 22)				
Age (years)	44.1	30	62	8.93
Users (*n* = 10)				
Age (years)	30.7	24	36	4.39
Age of youngest infant (months)	18.4	5	54	5.16
Residence duration (years)	4.45	2	17	4.54
Intercultural interpreters (*n* = 4)				
Age (years)	44.5	34	52	7.94

**Table 2:** Sample characteristics (*N* = 36)

Characteristics	*n*
Healthcare professionals	22
Sex	
Female	22
Nationality	
Swiss	18
Other	4
Profession	
Physician	4
Midwife	11
Nurse	2
Mothers’ counsellors	5
Professional experience	
1–2 years	0
3–10 years	8
More than 10 years	14
Frequency of care for allophone migrants	
One to several times per week	14
One to several times per month	6
One to several times per year	2
Users	10
Nationality	
Eritrean	6
Kosovar	3
Albanian	1
Family status	
Married	8
Single	1
Widowed	1
Mother tongue (multiple answers)	
Tigrinya	6
Albanian	4
Other	2
Mastering German language	
Not at all	0
Comprehends single words	1
Comprehends and talks single words	4
Comprehends and talks simple sentences	5
Residence permits	
N (Asylum seeker)	0
F (Provisionally admitted foreigners)	2
C (Settled foreign nationals)	1
B (Resident foreign nationals)	7
Intercultural interpreters	4
Sex	
Female	4
Nationality	
Eritrean	2
Kosovar	0
Albanian	1
Swiss	1
Professional experience	
1–2 years	2
3–10 years	2
More than 10 years	0
Frequency of interpreting assignments in maternity area	
One to several times per week	1
One to several times per month	2
One to several times per year	1

The 22 healthcare professionals, all female, were midwives, nurses, obstetricians, paediatricians and maternity counsellors. Half of them had a formal qualification for transcultural competences. Most were regularly involved in care or treatment of allophone mothers and families, who, in most cases, speak Tigrinya or Albanian.

The intercultural interpreters were two Eritrean, an Albanian and a Swiss female. They all had at least 1 year of experience translating on‐site and over the phone for pregnant women or young mothers.

### 2.4 Data collection

All interviews were conducted by the two main researchers (PO, GH). The Eritrean's users’ data were collected in an FGD led by a Tigrinya‐speaking moderator (FA). This FGD was observed by researchers assisted by a translator, all staying in the background. This allowed the researchers to follow the discussion and if necessary, to query or support the moderator. Because of scheduling difficulties, the Albanian‐speaking women attended three interviews, two single and one double, conducted by a researcher and an Albanian‐speaking translator. Audio recordings, which were transcribed directly into German, were made of all the interviews and field notes were made immediately after the interviews by the researchers. The healthcare professionals were interviewed in two FGDs and the intercultural interpreters had a single interview each, which were recorded and transcribed verbatim. The interviews lasted between 40 and 60 min and FGDs between 1 hr and 45 min and 2 hr.

### 2.5 Ethical considerations

Research on migrants is delicate because they are particularly vulnerable. Therefore, participants were fully informed in their mother tongue about the study before signing their consent to participate. Focus group members were informed of the need to preserve the confidentiality of group discussions. The ethical considerations were set out in detail in the study protocol (Origlia Ikhilor et al., 2017) and the ethics committee responsible issued a formal memorandum that no special Research Ethics Committee approval was necessary.

### 2.6 Data analysis

Two researchers (PO, GH) coded the interviews and created themes. Thematic analysis (Braun & Clarke, 2006) was then completed with ATLAS.TI® software 7.0.

### 2.7 Rigour

Rigour was assured by investigating this topic from three different perspectives. After every interview, participants were given a member check in the form of a verbal summary of the discussed points to confirm and correct their statements. Meetings among all researchers took place to discuss and agree on emerging findings. The analysis of user data required special attention because the researchers were at risk of interpreting and judging statements according to their own cultural background. To better understand users' cultural interpretive patterns, an Albanian and an Eritrean translator were included in the research team.

## 3 FINDINGS

Analysis revealed that the participants from all three groups commented on three overarching dimensions. Firstly, the dimension “circumstances and healthcare system” describes how communication is affected by the women's personal circumstances and the organization of the healthcare system. Secondly, “relations and interactions” encompasses communication issues between all participants. Finally, “adaptation of health services” which describes to what extent, given the quality of communication, the needs of the women corresponded with the health care given. From each group's perspective we categorized their responses into three themes (see the middle three columns of Table 3). From there we took the three themes for each dimension and produced “synthesized themes”. This enabled us to see what the main issues are and how the three group's perceptions coincided or diverged.

**Table 3:** Themes matrix

Dimension	Themes			Synthesized themes
	Users	Healthcare professionals	Intercultural interpreters	
Circumstances and healthcare system	Navigation through a complex system	Willingness to understand	Comprehension of circumstances	Challenge of understanding each other's world
Relations and interactions	Lack of rapport	Stripped down communication	Divergent expectations	Communication breakdown
Adaptation of health services	Lack of agency	Try or resign	Indispensable interpreters	Imposed health services

### 3.1 Challenge of understanding each other's world

The interviews suggested that all three groups genuinely tried to understand reality from the others’ perspective, despite the challenge to understand each other's different worlds and the unfamiliar system. Whereas intercultural interpreters sympathized with users because they knew the country of origin and were able to fall back on their own experience in an unfamiliar country, healthcare professionals sometimes lacked understanding of the women's social environment. The women on the other hand, lacked knowledge of the reproductive healthcare system.

For the allophone women, it was difficult to “navigate through the complex (healthcare) system”. Given Switzerland's highly differentiated system it was hard for them to know who to turn to, who was responsible for what and how to assess the usefulness of specific services:

The situation in which I was in taught me. Depending on the situation, I moved. I had no choice but to find my way around. (Albanian woman)

The women's present circumstances and social environment seemed to have a strong influence on communication. Their habits, expectations and values sometimes fundamentally differed from the judgements and habits of the healthcare professionals:

I did not want to go to the doctor and waited for the vomiting to stop. When the midwife visited she said: ‘You are pregnant, you have to go to the doctor’. But I do not have a family doctor. I was not ill, I did not need a doctor. (Eritrean woman)

The communication became particularly complex and demanding if social circumstances were very difficult, for example, their residence status was uncertain. The healthcare professionals showed considerable “willingness to understand”, that is, readiness to see and understand the women's circumstances and empathized with their stressful situation:

You think, “this woman has had her fourth child, she already knows how to do it”. But now, in a situation far away from home, the whole family in a small room in a transit home. [...] There is such agitation and insecurity in life – now even the things you know and have experience with become much more difficult and time‐consuming. (Midwife)

While for migrant women, the family often represented the most important resource in their unfamiliar situation, the professionals found the relatives sometimes more of a handicap. They found it difficult to connect with them and rarely used them as a resource—at most only as ad hoc interpreters:

It is partly a clan, they live in a house of three, four families who know each other. Yes, they take care of each other very well, but I find it difficult to approach them. Or, as noted in the referral report: “Wishes no counselling”. (Maternity counsellor)

Although interpreters and healthcare professionals recognized the disorientation of some migrant women interpreters found it easier to “comprehend the circumstances” of allophone migrant women. They explained that existential, rather than health, problems could overshadow a consultation. These situations were frustrating for the interpreters as they were unable to give a solution in the scope of their task:

Asylum seekers put great hope in every meeting. The person they speak to is very, very important to them. But sometimes that person disappoints. And then it's solely that person's fault ‐ mine, the translator’s, or the healthcare professional’s, because they did not help. (Albanian interpreter)

### 3.2 Communication breakdown

Both healthcare professionals and allophone migrant women tried to understand each other but were unable to, not through lack of effort but it was just the completely different languages spoken by the parties. This led to communication being reduced to a bare minimum and thus, insufficient to inform women about treatment and adequately address their expectations and needs.

Some women reported very good care experiences despite the language barriers and that professionals were strongly committed to them and considered their situation. Others reported a “lack of rapport”, for example, that the relationship between them and the healthcare providers was deficient. Any perceived dominance of the professional over the lay person in their relationship was intensified by the language barriers. Women described this as a relationship gap between themselves and the healthcare professionals:

I did not understand what they then discussed among themselves. To this day, I have no explanation why all this had to be done [a termination of pregnancy with complications]. […] They could have treated me as a human. (Eritrean woman)

The women felt offended if procedures and interventions were inadequately or not explained. Some women described situations where they assumed they were being discriminated against:

The problem was that we were informed that the unborn child was sick. And in the end, it was born healthy. We did not understand why [the doctor told us the child was sick], we then thought, maybe it was because we have a different skin colour ‐ that's what we think. (Eritrean woman)

In this case it is probable that the woman did not understand the concept of probabilities associated with diagnosis.

In some cases following a bad experience, the women avoided further consultations. Healthcare professionals and interpreters made no direct statements on discriminatory behaviour but revealed some expectations about the women and their families’ integration efforts:

There are demands [from the women]. But there are no efforts to integrate and no efforts to communicate. This is where I reach my limit. (Midwife)

The healthcare professionals repeatedly used various forms of non‐verbal communication, such as pictures or communication with gestures. A touch or a shared laugh could help build trust, but without interpreting verbal “communication was stripped down” or simplified. Only very limited communication and information exchange was possible. This clouded communication, although the degree of misunderstanding was not always recognized:

I often had the feeling that we understood each other quite well just by talking with hands and feet. Then when the woman comes again, she does something quite differently from what I told her to do. [...] Did I really say so? And she says, "You said." And afterwards I think: My word! Did I say that? (Maternity counsellor)

Intercultural interpreters pointed out that it is important to use simple language and that enough time is taken. Moreover, interpreters and professionals commented that the interpreters’ limited availability meant there was a tendency to pack as much information into one consultation as possible, which naturally often overwhelmed the women:

What I find difficult after having organized an interpreter any number of different people come: nurses, the midwife, the paediatrician, the obstetrician… How can the woman take it all in such a short time? But we're not allowed to have an interpreter twice, so we’re all packed into one meeting. (Nurse)

In some situations, professionals were unable to understand the needs and expectations of women and focussed instead on their functional treatment. Even with professional intercultural translation they found themselves talking at cross purposes. Interpreters witnessed how service users’ and healthcare professionals’ “expectations diverged” sometimes:

Perhaps we could show a little more patience and listen to these worries. Because the woman really needed somebody to tell her worries. And this was such a case, she had no one and suddenly someone came [healthcare professional] and she expected her problems to be heard. (Albanian Interpreter)

### 3.3 Imposed health services

A lack of communication sometimes caused healthcare provision to seem imposed in an authoritarian way on the women and unresponsive to their real needs.

The women reported situations where an intervention contradicted their expectations. To some women, especially Eritreans, intervention‐free pregnancy or birth was important. The providers were aware that some interventions may have contradicted the women's value systems and that discussing certain topics was perceived as impertinent:

I expected the contractions to come naturally and to wait. I wanted to experience how that is, the contractions, as in our home. (Eritrean woman)

Often, in my consultations, I’d like to discuss the results of an examination, but the pregnant women only want to know: “Baby good or not”? Then, you do not even know where to start and where to stop. A husband once, accused me of psychological terror because I informed them about the first‐trimester test, including the risk of Down Syndrome and he did not understand that. (Obstetrician)

The women felt a “lack of agency” and that they were at the mercy of the providers. In many situations, they did not understand the reasons of the medical intervention, so they could not give informed consent. This made them to feel helpless and concerned. Long‐lasting bad feelings sometimes lead them to renunciate further care:

The physician explained the operation [caesarean section] as well as possible. I just said yes, yes, I couldn’t say anything else. For example, I couldn’t ask, "Can I wait another week, can I wait until my due term?". (Eritrean woman)

Healthcare professionals found themselves caught between “trying and resigning” that is, having to decide whether to try their hardest to communicate or come to terms with the situation. Often, professionals resigned themselves to frustrating communication and comforted themselves with the fact that the mother and baby's health was fine, and that Swiss health care is almost certainly better than that in the woman's country of origin:

We always want [the women] to understand us. We want to be on the same basis. But sometimes it's not the same there [in their home country]. Sometimes here they think: “Wow, that's crazy ...! They say: that's different with us.” And of course, if we have our approach, we are frustrated because we cannot talk to them. […] I find it very difficult to find a balance and not have such an awfully guilty conscience just because you cannot understand each other. (Obstetrician)

Non‐involvement in the decision processes triggered a range of negative sentiments in women, from mild concern to existential anxiety, especially in emergencies. Emergencies were a big strain for all involved, including interpreters:

We had to bring a woman in for an emergency operation. I felt like I was raping her, terrible, violating, crossing a line, forced to use some kind of catheter without being able to explain her the why and wherefore. (Midwife)

It was critical for me and the baby. They had to do something. I didn’t understand anything […] I just signed blindly. I did not know what happened to me, what happened to the baby. Is my baby still alive or has it died? I had no idea what help I should get, I was scared, I started to cry. (Albanian woman)

All interviewed groups, but most of all the migrant women, found that “interpreting services were indispensable” to achieving appropriate maternity care:

Every person who comes for treatment has the right to understand what is being done to him or her. (Eritrean interpreter)

## 4 DISCUSSION

This study assessed the communication experiences during maternity care for allophone migrant women in the German speaking part of Switzerland. It confirmed that language barriers cause problems for women in finding appropriate care and understanding health information and counselling. Specifically, informed consent for interventions was not possible when communication was limited to non‐verbal gestures and insufficient vocabulary. Access to an interpreter's services was desired, but often not possible. Taking different perspectives allowed us to identify shared experiences and divergent ones, thus highlighting the magnitude of communicative barriers.

Both users and providers struggled to understand each other's circumstances. Their frustrations, however, were centred on different issues. Allophone women were often disorientated in the Swiss healthcare system, which is highly complex and typified by a variety of different providers. They sometimes expected help not only for health issues but also for many other concerns including unsolved existential problems even though healthcare professionals did not have the authority to directly respond to social or legal issues. Depending on their residence status, migrants access healthcare services in different ways: permanent foreign residents must fend for themselves while refugees are supported—to a lesser or greater extent—by local authorities (Origlia Ikhilor et al., 2017). A study on how Swiss asylum centres organize healthcare provision showed that sexual and reproductive healthcare needs of women asylum seekers are not sufficiently addressed and there is a lack of translation and gender sensitive approach of women (Cignacco et al., 2018). There is an urgent need to better target health care at allophone women's needs and women need guidance through the Swiss maternity healthcare system to be able to make informed decisions.

The providers were challenged when trying to grasp the social system of migrant women. Although for the women, the family represents an important moral and practical crutch, healthcare professionals did not always succeed in integrating relatives appropriately into the care process and often perceived them as a hindrance. Other studies have described relatives sometimes feeling unwanted, highly worried, and often lacking adequate information too (Bihr & Kaya, 2014). The patterns of interaction of Swiss healthcare professionals with individualistic socialization and migrants with sociocentric socialization often fundamentally differ (Domenig, 2007). That said, in most cases the social network of migrant women is often restricted compared with how it was in their homeland. Self‐selected reference persons should be addressed actively by healthcare professionals. They need to be informed comprehensively and their fears and concerns also taken into account (Bihr & Kaya, 2014). The efficient involvement of the family is a resource and a protective factor. Moreover, intercultural interpreters can support healthcare professionals in assessing the migrant women's family system and identifying available intra‐family resources.

Breakdowns in communication were frustrating for all involved. The allophone women reported some discriminatory behaviour from healthcare professionals. Although providers and interpreters made no direct statements about this, the professionals did express expectations about the integration efforts of women and families. It is therefore conceivable that expectations were said unconsciously and led to users’ negative feelings. Discrimination and professionals’ unintended, unfavourable attitudes naturally discourage women from engaging in consultations (Origlia, Jevitt, Sayn‐Wittgenstein, & Cignacco, 2017). Transcultural competence development can help to sensitize professionals to their behaviour and unintentional discrimination. Essentially, transcultural competence in healthcare is the ability to interact effectively with migrants across cultural boundaries (Domenig, 2007). To do this, professionals must first learn to perceive their own lifeworld through self‐reflection. Together with background knowledge for example, about migration processes, they can better classify and understand migrants’ circumstances and environment. Finally, narrative empathy allows professionals an appreciative, respectful attitude towards migrants. By placing narration at the centre of treatment, carers can reflect on their own prejudices and discriminatory behaviour (Domenig, 2007). In our study, almost half of the interviewed professionals had completed training in transcultural competence. In practice, healthcare professionals generally feel less proficient in intercultural tasks than medical skills (Hudelson, Perron, & Perneger, 2011).

According to the interviews the most common strategy to overcome language barriers was communication with gestures. Consequently, health education or promotion and profound counselling to facilitate informed choices was not possible, thus misunderstandings and mistreatments sometimes occurred. Women became confused by the limited communication and had many unanswered questions even after the treatment was completed. The lack of communication often led to loss of trust on the part of the women, while the healthcare professionals where often unaware of subsequent problems or lasting bad memories of maternity care. Communication with gestures seemed to some extent to be approved by the healthcare professionals, although they were aware of its limitations. Moreover, if mother and baby could be healthily discharged, the result of their treatment was considered sufficient. It was unclear to what extent the professionals recognize the true implications inadequate communication. Possibly they tend to resign themselves to the situation because they have no other option.

Even with involvement of intercultural interpreters, communication between healthcare professionals and allophone women could be inefficient, especially if the expectations of a consultation differed. Sometimes too many professionals were included in a consultation or too much information given at once, which overwhelmed the women. Maternity care counselling on health education and promotion can risk hitting the women with a wall of information that does not apply to individual needs and concerns. It is much more efficient when professionals are allowed to have a solid and continuous relationship with women (Sanders, Hunter, & Warren, 2016).

Emergency situations were particularly stressful for all involved. Professionals were faced with a dilemma: they needed to give comprehensive information and at the same time they had to take immediate therapeutic measures to safeguard mother and child. Obstetric emergencies, neonatal complications and poor quality of provider interactions can cause great anxiety and even traumatize women because they cannot assess the extent of the danger to themselves and their child (Simpson & Catling, 2016). Forcing people to sign a consent form without understanding its content is an extreme measure that raises ethical questions. While professionals argue that they are not allowed to intervene without the signed document, the signature should confirm that the user understands the situation. Interpretation via telephone is a possible solution in emergency situations (Swiss National Ethical Commission [Nationale Ethikkommission], 2017).

The healthcare professionals were sometimes stretched to their limits and subject to many restrictions that prevented them from responding flexibly to the women's needs. These restrictions included the prescribed routine of prenatal examinations, internal guidelines on obstetric interventions, tight schedules, and austerity measures. The complexity of the situations did not allow them to directly solve the women's and their families’ problems, or to give holistic care in accordance to the cultural values of their people. Some treatments seemed imposed on women who would have preferred a natural approach, for example, spontaneous onset of labour, no episiotomy or no caesarean section. From the individual reports given by our sample it cannot be judged if such interventions were medically justified. However, in recent decades, there has been a dramatic increase in obstetric interventions. This oversupply is recognized as a public health problem that exposes women and newborns to unnecessary immediate and long‐term health problems (Bingham, Ruhl, & Cockey, 2013). Healthcare professionals need agency to accommodate individual and cultural preferences in the physiological processes of pregnancy and childbirth. This can be difficult if obstetric standards and guidelines do not include real alternatives.

### 4.1 Strengths and Limitations

In several meetings a group of researchers (PO, GH, EK, JP, EC) reviewed topics and determined how well the themes matched the coded data. Carefully reviewing the findings and interviews as a group allowed the researchers to analyse and interpret the data to generate agreed on interpretations and eliminate misleading ones (Braun & Clarke, 2006).

Despite different perspectives being used in this study the sample of service users was relatively small and limited to only two population groups with different socio‐cultural characteristics and migration histories. Therefore, conclusions about other allophone migrants are only possible to a limited extent.

The predefined exclusion criteria had to be relaxed during the recruitment, for example, few women were able to have a simple conversation in German. Nevertheless, their data gave insight into communicative challenges about their experiences. Furthermore, during the interviews it became apparent that some mothers had had complications during their last birth but were willing to talk about their experiences anyway. Therefore, they were not excluded retrospectively.

All the Eritrean women were recruited through a single person who was also involved as an intercultural interpreter in the obstetric consultation of some of them. Moreover, this interpreter was present at the FGD and translated for the researchers. The participation of a known person may have caused a loyalty conflict among the women and triggered some socially desired answers.

In the foreign language interviews and FGD some information was not immediately and fully accessible to the researchers. We therefore put significant effort into instructing the interpreters/moderator so that they had a comprehensive understanding of the method. No back translation of the Tigrinya and Albanian transcripts was. However, when uncertainties arose, the researchers could compare the transcript and the recorded verbal translation. Despite these limitations, there is methodological consensus that useful knowledge can be gained (Kruse, 2012; Lauterbach, 2014).

## 5 CONCLUSION

There is a significant range of communication barriers in maternity health care provision that lead to adverse short‐ and long‐term results. Measures to dismantle barriers include first and foremost, secured and financed intercultural interpreting services. As these are not always available on site for example, in emergency situations, therefore telephone services must be considered. Only through interpretation can quality treatment be achieved and legally required informed consent obtained. Lack of agency and understanding in emergencies can be traumatic for women and is stressful for healthcare professionals too. Awareness must be raised that communication with gestures is not enough.

Healthcare professionals’ transcultural competencies must be continuously developed and integrated into education and practice. Continuous reflection may also raise awareness of discriminatory behaviour. Training in the use of plain language and avoidance of information overload would lower language barriers and make translation easier. Their advice should focus on women's concerns and continuity of care should be prioritized.

Healthcare settings for allophone mothers must be more flexible and offer care options that are in accordance with the women's cultural values. Obstetric guidelines, however, may not offer the option of a low‐intervention, more natural approach. We would therefore recommend that guidelines should be counterchecked to see if they can be adapted.

Furthermore, migrant women need support navigating an unknown healthcare system. This is not just relevant to pregnant women. To encourage integration, women should be given health system information as early as possible and given the opportunity to process this information with trained peers, integration officers or healthcare professionals.

In summary, better communication during maternity care cannot only be achieved through better individual attitude or professional skills but it also needs appropriate conditions at institutional and policy levels. Improvement will not only help achieve better mother and child health but also help engage healthcare professionals in their challenging task.

## CONFLICT OF INTEREST

The authors declare no conflict of interest.

## AUTHORS CONTRIBUTIONS

Made substantial contributions to conception and design, or acquisition of data, or analysis and interpretation of data.

## References

[jan14093-bib-0001] Bihr, S. , & Kaya, B. (2014). Verletzliche Personen in der Gesundheitsversorgung. Erfahrungen aus der Sicht von Personen mit Migrationshintergrund. Wabern‐Bern, Switzerland: SRK.

[jan14093-bib-0002] Bingham, D. , Ruhl, C. , & Cockey, C. D. (2013). Don't Rush Me … Go the Full 40: AWHONN's public health campaign promotes spontaneous labor and normal birth to reduce overuse of inductions and cesareans. The Journal of Perinatal Education, 22(4), 189–193. 10.1891/1058-1243.22.4.189 24868131 PMC4010855

[jan14093-bib-0003] Boerleider, A. W. , Francke, A. L. , Mannien, J. , Wiegers, T. A. , & Deville, W. L. (2013). "A mixture of positive and negative feelings": A qualitative study of primary care midwives' experiences with non‐western clients living in the Netherlands. International Journal of Nursing Studies, 50(12), 1658–1666. 10.1016/j.ijnurstu.2013.04.009 23721761

[jan14093-bib-0004] Braun, V. , & Clarke, V. (2006). Using thematic analysis in psychology. Qualitative Research in Psychology, 3(2), 77–101. 10.1191/1478088706qp063oa

[jan14093-bib-0005] Bundesamt für Gesundheit (2012). Gesundheit der Migrantinnen und Migranten in der Schweiz, Wichtigste Ergebnisse des zweiten Gesundheitsmonitorings der Migrationsbevölkerung in der Schweiz, 2010. Bern, Switzerland: BAG.

[jan14093-bib-0006] Bundesamt für Gesundheit . (2013). Nationales Programm Migration und Gesundheit. Bilanz 2008–13 und Schwerpunkte 2014–17. Bern, Switzerland: BAG.

[jan14093-bib-0007] Bundesamt für Statistik . (2018a). Lebendgeburten nach Geschlecht und Geburtenfolge des Kindes, Staatsangehörigkeit und Zivilstand der Mutter sowie Altersklasse der Eltern. Retrieved from https://www.bfs.admin.ch/

[jan14093-bib-0008] Bundesamt für Statistik . (2018b). Personen, die 3, 2, 1 oder keine Landessprache beherrschen. Retrieved from https://www.bfs.admin.ch/

[jan14093-bib-0009] Cignacco, E. , zu Sayn‐Wittgenstein, F. , Sénac, C. , Hurni, A. , Wyssmüller, D. , Grand‐Guillaume‐Perrenoud, J. A. , & Berger, A. (2018). Sexual and reproductive healthcare for women asylum seekers in Switzerland: A multi‐method evaluation. BMC Health Services Research, 18(1), 712 10.1186/s12913-018-3502-2 30217153 PMC6137714

[jan14093-bib-0010] de Graaf, J. P. , Steegers, E. A. , & Bonsel, G. J. (2013). Inequalities in perinatal and maternal health. Current Opinion in Obstetrics and Gynecology, 25(2), 98–108. 10.1097/GCO.0b013e32835ec9b0 23425665

[jan14093-bib-0011] Domenig, D. (2007). Transkulturelle Kompetenz. Lehrbuchbuch für Pflege‐, Gesundheits‐ und Sozialberufe. Bern, Switzerland: Hans Huber.

[jan14093-bib-0012] Gehrig, M. , Caldéron, R. , Guggisberg, J. , & Gardiol, L. (2012). Einsatz und Wirkung von interkulturellem Übersetzen in Spitälern und Kliniken. Bern, Switzerland: Büro für arbeits‐ und sozialpolitische Studien BASS Retrieved from https://www.buerobass.ch/fileadmin/Files/2012/BAG_2012_Einsatz_Wirkung_ikUe_Schlussbericht.pdf

[jan14093-bib-0013] Higginbottom, G. , Bell, A. S. , Arsenault, J. , & Pillay, J. (2012). An integrative review of experiences of maternity services for immigrant women in Canada. Diversity & Equality in Health & Care, 9(4), 253–266.

[jan14093-bib-0014] Hudelson, P. , Perron, N. J. , & Perneger, T. (2011). Self‐assessment of intercultural communication skills: A survey of physicians and medical students in Geneva, Switzerland. BMC Medical Education, 11, 63 10.1186/1472-6920-11-63 21884609 PMC3175208

[jan14093-bib-0015] Interpret . (2017). Interkulturelles Dolmetschen und Vermitteln. Retrieved from http://www.inter-pret.ch/

[jan14093-bib-0016] Keygnaert, I. , Guieu, A. , Ooms, G. , Vettenburg, N. , Temmerman, M. , & Roelens, K. (2014). Sexual and reproductive health of migrants: Does the EU care? Health Policy, 114, 215–225. 10.1016/j.healthpol.2013.10.007 24268324

[jan14093-bib-0017] Keygnaert, I. , Ivanova, O. , Guieu, A. , Van Parys, A.‐S. , Leye, E. , & Roelens, K. (2016). What is the evidence on the reduction of inequalities in accessibility and quality of maternal health care delivery for migrants? A review of the existing evidence in the WHO European Region. Copenhagen, Denmark: WHO. 27786434

[jan14093-bib-0018] König, C. , & Pehlke‐Milde, J. (2010). Bestandesaufnahme des Betreuungs-, Beratungs- und Unterstützungsangebots für Wöchnerinnen in der Schweiz : Schlussbericht. Bern, Switzerland: Bundesamts für Gesundheit (BAG).

[jan14093-bib-0019] Krueger, R. A. , & Casey, M. A. (2009). Focus groups : A practical guide for applied research, 4th ed Thousand Oaks, CA: Sage.

[jan14093-bib-0020] Kruse, J. (2012). Qualitative Interviewforschung in und mit fremden Sprachen: Eine Einführung in Theorie und Praxis. Weinheim, Germany: Beltz Juventa.

[jan14093-bib-0021] Kurth, E. (2013). FamilyStart beider Basel – ein koordinierter Betreuungsservice für Familien nach der Geburt. Hebamme.ch 7/8, 35–37.

[jan14093-bib-0022] Lauterbach, G. (2014). Dolmetscher/inneneinsatz in der qualitativen Sozialforschung. Zu Anforderungen und Auswirkungen in gedolmetschten interviews. Forum: Qualitative Sozialforschung, 15, 2.

[jan14093-bib-0023] Levesque, J. F. , Harris, M. F. , & Russell, G. (2013). Patient‐centred access to health care: Conceptualising access at the interface of health systems and populations. International Journal for Equity in Health, 12, 18 10.1186/1475-9276-12-18 23496984 PMC3610159

[jan14093-bib-0024] Merten, S. , & Gari, S. (2013). Die reproduktive Gesundheit der Migrationsbevölkerung in der Schweiz und anderen ausgewählten Aufnahmeländern. Eine Zusammenfassung der Literatur 2006–2012. Basel, Switzerland: BAG.

[jan14093-bib-0025] Nationale Ethikkommmission and (Commission nationale d’éthique dans le domaine de la médecine humaine CNE) . (2017). Migrants allophones et système de soins. Enjeux éthiques de l'interprétariat communautaire. NEK/CNE. Retrieved from http://www.nek-cne.ch/

[jan14093-bib-0026] Navarra, K. , & Liewald, K. (2017). Health guide to Switzerland. In migesplus.ch (Ed.). Bern, Switzerland: Swiss Red Cross & Federal Office of Public Health (FOPH).

[jan14093-bib-0027] Origlia Ikhilor, P. , Hasenberg, G. , Kurth, E. , Stocker Kalberer, B. , Cignacco, E. , & Pehlke‐Milde, J. (2017). Barrier‐free communication in maternity care of allophone migrants: BRIDGE study protocol. Journal of Advanced Nursing, 74(2), 472–481. 10.1111/jan.13441 28833465

[jan14093-bib-0028] Origlia, P. , Jevitt, C. , Sayn‐Wittgenstein, F. Z. , & Cignacco, E. (2017). Experiences of antenatal care among women who are socioeconomically deprived in high‐income industrialized countries: An integrative review. Journal of Midwifery & Women's Health, 62(5), 589–598. 10.1111/jmwh.12627 28763167

[jan14093-bib-0029] Pelaez, S. , Hendricks, K. N. , Merry, L. A. , & Gagnon, A. J. (2017). Challenges newly‐arrived migrant women in Montreal face when needing maternity care: Health care professionals' perspectives. Global Health, 13(1), 5 10.1186/s12992-016-0229-x 28122630 PMC5264303

[jan14093-bib-0030] Sanders, J. , Hunter, B. , & Warren, L. (2016). A wall of information? Exploring the public health component of maternity care in England. Midwifery, 34, 253–260. 10.1016/j.midw.2015.10.013 26608787

[jan14093-bib-0031] Sentell, T. , Chang, A. , Ahn, H. J. , & Miyamura, J. (2016). Maternal language and adverse birth outcomes in a statewide analysis. Women and Health, 56(3), 257–280. 10.1080/03630242.2015.1088114 26361937 PMC4868388

[jan14093-bib-0032] Simpson, M. , & Catling, C. (2016). Understanding psychological traumatic birth experiences: A literature review. Women Birth, 29(3), 203–207. 10.1016/j.wombi.2015.10.009 26563636

[jan14093-bib-0033] Tong, A. , Craig, J. , & Sainsbury, P. (2007). Consolidated criteria for reporting qualitative research (COREQ): A 32‐item checklist for interviews and focus groups. International Journal for Quality in Health Care, 19(6), 349–357. 10.1093/intqhc/mzm042 17872937

[jan14093-bib-0034] Vermeir, P. , Vandijck, D. , Degroote, S. , Peleman, R. , Verhaeghe, R. , Mortier, E. , … Vogelaers, D. (2015). Communication in healthcare: A narrative review of the literature and practical recommendations. International Journal of Clinical Practice, 69(11), 1257–1267. 10.1111/ijcp.12686 26147310 PMC4758389

[jan14093-bib-0035] Vos, A. A. , Posthumus, A. G. , Bonsel, G. J. , Steegers, E. A. , & Denktas, S. (2014). Deprived neighborhoods and adverse perinatal outcome: A systematic review and meta‐analysis. Acta Obstetricia Et Gynecologica Scandinavica, 93(8), 727–740. 10.1111/aogs.12430 24834960

[jan14093-bib-0036] Witzel, A. (2000). Das problemzentrierte interview. Forum Qualitative Forschung, 1(1).

