# Supplementary material for: Communication barriers in maternity care of allophone migrants: Experiences of women, healthcare professionals, and intercultural interpreters
Source: J Adv Nurs. 2019 Jun 21;75(10):2200–10. doi: 10.1111/jan.14093 (PMC6852258; doi:10.1111/jan.14093)
Supplement: Supplementary file 1 [file JAN-75-2200-s001.docx]

**COREQ Checklist (Tong, Craig, & Sainsbury, 2007)**

| **Topic** | **Item No.** | **Guide Questions/Description** | **Reported on Page No.** |
| --- | --- | --- | --- |
| **Domain 1: Research team and reflexivity** | | | |
| *Personal characteristics* | | | |
| Interviewer/facilitator | 1 | Which author/s conducted the interview or focus group? | Method, p 8 |
| Credentials | 2 | What were the researcher’s credentials? E.g. PhD, MD | Title page, authors details |
| Occupation | 3 | What was their occupation at the time of the study? | Title page, authors details |
| Gender | 4 | Was the researcher male or female? | All female, title page, authors details |
| Experience and training | 5 | What experience or training did the researcher have? | See comments |
| *Relationship with participants* | | |  |
| Relationship established | 6 | Was a relationship established prior to study commencement? | Study protocol (Origlia Ikhilor et al., 2017) pp 477-8 |
| Participant knowledge of the interviewer | 7 | What did the participants know about the researcher? e.g. personal goals, reasons for doing the research | Origlia et al. 2017, p 477 |
| Interviewer characteristics | 8 | What characteristics were reported about the inter viewer/facilitator? e.g. Bias, assumptions, reasons and interests in the research topic | Origlia et al. 2017, pp 478, 479 |
| **Domain 2: Study design** | | | |
| *Theoretical framework* | | | |
| Methodological orientation and Theory | 9 | What methodological orientation was stated to underpin the study? e.g. grounded theory, discourse analysis, ethnography, phenomenology, content analysis | Method, p 7 and Origlia et al. 2017, pp 475, 478 |
| *Participant selection* | | | |
| Sampling | 10 | How were participants selected? e.g. purposive, convenience, consecutive, snowball | Method, p. 7 and Origlia et al. 2017, pp 475-477 |
| Method of approach | 11 | How were participants approached? e.g. face-to-face, telephone, mail, email | Origlia et al. 2017, p 477 |
| Sample size | 12 | How many participants were in the study? | Method, p 7 |
| Non-participation | 13 | How many people refused to participate or dropped out? Reasons? | N/A, see comments |
| *Setting* | | |  |
| Setting of data collection | 14 | Where was the data collected? e.g. home, clinic, workplace | Origlia et al. 2017, p 477 |
| Presence of non-participants | 15 | Was anyone else present besides the participants and researchers? | Method, p. 8 and Origlia et al. 2017, p 477 |
| Description of sample | 16 | What are the important characteristics of the sample? e.g. demographic data, date | Method, p. 7,8, table 1 and 2 and Origlia et al. 2017, pp 475-7 |
| *Data collection* | | | |
| Interview guide | 17 | Were questions, prompts, guides provided by the authors? Was it pilot tested? | Origlia et al. 2017, p 477 |
| Repeat interviews | 18 | Were repeat interviews carried out? If yes, how many? | N/A |
| Audio/visual recording | 19 | Did the research use audio or visual recording to collect the data? | Method, p.8 and Origlia et al. 2017, p 478 |
| Field notes | 20 | Were field notes made during and/or after the interview or focus group? | Method, p 8 |
| Duration | 21 | What was the duration of the inter views or focus group? | Method, p 8 |
| Data saturation | 22 | Was data saturation discussed? | see comments |
| Transcripts returned | 23 | Were transcripts returned to participants for comment and/or correction? | No. Method, p. 9, Origlia et al. 2017, pp 477-8 and see comments |
| Data analysis | | | |
| Number of data coders | 24 | How many data coders coded the data? | Method, p 8. |
| Description of the coding tree | 25 | Did authors provide a description of the coding tree? | N/A, the coding tree is not available in English |
| Derivation of themes | 26 | Were themes identified in advance or derived from the data? | Results |
| Software | 27 | What software, if applicable, was used to manage the data? | Results |
| Participant checking | 28 | Did participants provide feedback on the findings? | Results |
| *Reporting* |  |  |  |
| Quotations presented | 29 | Were participant quotations presented to illustrate the themes/findings? Was each quotation identified? e.g. participant number | Results, and see comments |
| Data and findings consistent | 30 | Was there consistency between the data presented and the findings? | Strengths and limitation, p. 23 |
| Clarity of major themes | 31 | Were major themes clearly presented in the findings? | Results, table 3 |
| Clarity of minor themes | 32 | Is there a description of diverse cases or discussion of minor themes? | Results, table 3 |

**Comments:**

5: Researcher 1 and 2 had extensive midwifery, obstetrics and research experience. Researchers 3, 4 and 5 were senior researcher with several years of qualitative research and publication experience. Researcher 1 and 4 acted as transcultural experts, expert four’s origin corresponded to one part of the migrant’s study population. Researcher 4, as medical doctor, had extensive research experience too.

13: As the recruitment of participants was done by third parties e.g. contact persons and institutions, the exact number of requests and refusals was not systematically documented. It was more difficult to recruit users, in contrast to professionals and intercultural interpreters. Recruiting migrants through contact persons of the same origin was the most efficient strategy, while recruiting through hospitals or institutions was largely unsuccessful.
Documented reasons for the non-participation of users were: not interested; speak good German; health reasons; still pregnant; lost contact; travelled to their homeland; husband does not want it; husband agrees, but woman does not want; works again; would agree, but only if a job / an employment can be arranged.

22: Due to the available financial resources for the study the number of the interviews was predefined. The triangulation process showed that all three participant groups commented on similar themes and the rich data allowed plausible conclusions.

23: For validation a member check was conducted, but transcripts were not returned to participants.

28: The quotation of the focus group discussion could not completely be assigned to the individuals. For this reason, the quotes only provided information on group assignment, profession and origin.

**References**

Origlia Ikhilor, P., Hasenberg, G., Kurth, E., Stocker Kalberer, B., Cignacco, E., & Pehlke-Milde, J. (2017). Barrier-free communication in maternity care of allophone migrants: BRIDGE study protocol. *Journal of advanced nursing*, n/a-n/a. doi:10.1111/jan.13441

Tong, A., Craig, J., & Sainsbury, P. (2007). Consolidated criteria for reporting qualitative research (COREQ): a 32-item checklist for interviews and focus groups. *International Journal for Quality in Health Care, 19*(6), 349-357. doi:10.1093/intqhc/mzm042
